# Dental Exams Among Adults Aging With Intellectual and Developmental Disabilities in Different Living Arrangements

**DOI:** 10.1093/geroni/igaf122.372

**Published:** 2025-12-31

**Authors:** Elisabeth Stam, Jeffrey Stokes, Danielle Waldron

**Affiliations:** University of Massachusetts Boston, Boston, Massachusetts, United States; University of Massachusetts Boston, Boston, Massachusetts, United States; Stonehill College, North Easton, Massachusetts, United States

## Abstract

**Background and Objectives:**

Oral health is crucial for overall health and well-being across the lifespan. Receiving routine dental examinations and regular checkups with advancing age is paramount, especially for vulnerable populations such as adults with intellectual and developmental disabilities (I/DD). The dental health needs of adults with I/DD are often overlooked and barriers exist impeding their engagement with dental care services.

**Research Design and Methods:**

We analyzed 8 panel data waves (2013-2014 through 2021-2022) from the National Core Indicators In-Person Survey (NCI-IPS), which is a national survey of adults with I/DD (18+) who receive state services (N = 133,225 observations from 49 states). We performed multilevel logistic regression with models investigating whether adults with I/DD underwent dental exams and how dental services utilization trends differed by living situation.

**Results:**

Results demonstrated that adults with I/DD living in intermediate care facilities (ICF) settings are the most likely to receive a dental exam, while individuals with I/DD residing with their families are the least likely, but this divide lessened with age. Adults living with more severe I/DD have a lower likelihood of receiving a dental exam. Engaging in activities, community involvement, having friends, and having a job were significantly associated with an increased likelihood of receiving dental exams.

**Discussion and Implications:**

Findings indicate that adults with I/DD access dental care in varying degrees based on living arrangement, I/DD level, and social and community ties. Further research is necessary to understand the factors that contribute to promoting dental services utilization among this population.

